# Increased Serum IL 6 Associated With Mortality and Cognitive Impairment in Elderly Brazilians

**DOI:** 10.1093/geroni/igaa057.396

**Published:** 2020-12-16

**Authors:** Marcelo Caldeira, Milena Brunialti, Reinaldo Salomão, Clineu Almada Filho, Maysa Cendoroglo

**Affiliations:** 1 Universidade federal de São Paulo, SAO PAULO, Sao Paulo, Brazil; 2 UNIVERSIDADE FEDERAL DE SÃO PAULO, SAO PAULO, Brazil; 3 UNIVERSIDADE FEDERAL DE SÃO PAULO, SAO PAULO, Sao Paulo, Brazil

## Abstract

Immunological and inflammatory changes are gaining importance in aging as they are associated with functional limitations and mortality. Chronic inflammation associated with aging (Inflammaging) is a systemic and subclinical condition, characterized by changes in the levels of interleukins such as IL1, IL4, IL6, IL8, IL10 and TNF alpha, associated with genetic, physiological and environmental factors, whose importance is to be directly associated with morbidity and mortality in the elderly. Objective: To evaluate, through a longitudinal study, the relationship between chronic inflammation associated with aging and possible outcomes, such as cognitive changes and mortality in independent oldest old adults. Methods: were evaluated 201 elderly, aged 80 years or older, community residents, with preserved cognition, without acute diseases and with controlled chronic diseases. In a 02 years of interval, laboratory collections of inflammatory markers (IL 1, IL 4, IL6, IL10, TNF alpha and CRP) were performed and outcomes such as cognitive impairment and deaths were evaluated. Results: There was a correlation between increased serum IL6 and cognitive impairment, in the group of women (p-value = 0.008) and in the group All (p-value = 0.022). In the group of men, there was a significant difference between the increase in IL6 values ​​(p-value = 0.028) and CRP (p-value = 0.016) in relation to deaths. Conclusion: The results of this longitudinal study showed and confirmed the positive association between changes in inflammatory markers such as IL6 and outcomes such as cognitive impairment and mortality also in elderly Brazilians.

